# Inner Harmony as an Essential Facet of Well-Being: A Multinational Study During the COVID-19 Pandemic

**DOI:** 10.3389/fpsyg.2021.648280

**Published:** 2021-03-26

**Authors:** David F. Carreno, Nikolett Eisenbeck, José Antonio Pérez-Escobar, José M. García-Montes

**Affiliations:** ^1^Department of Psychology, University of Almería, Almería, Spain; ^2^Department of Personality, Evaluation and Psychological Treatment, Faculty of Psychology, University of Seville, Seville, Spain; ^3^Chair of History and Philosophy of Mathematics, Department of Humanities, Social and Political Sciences, Eidgenössische Technische Hochschule (ETH) Zürich, Zurich, Switzerland

**Keywords:** existential positive psychology, PERMA, mature happiness, inner harmony, psychological distress, COVID-19

## Abstract

This study aimed to explore the role of two models of well-being in the prediction of psychological distress during the COVID-19 pandemic, namely PERMA and mature happiness. According to PERMA, well-being is mainly composed of five elements: positive emotions, engagement, relationships, meaning in life, and achievement. Instead, mature happiness is understood as a positive mental state characterized by inner harmony, calmness, acceptance, contentment, and satisfaction with life. Rooted in existential positive psychology, this harmony-based happiness represents the result of living in balance between positive and negative aspects of one's life. We hypothesized that mature happiness would be a more prominent protective factor during the present pandemic than the PERMA composite. A total of 12,203 participants from 30 countries responded to an online survey including the Depression Anxiety Stress Scale (DASS-21), the PERMA-Profiler, and the Mature Happiness Scale-Revised (MHS-R). Confirmatory factor analyses indicated that PERMA and mature happiness were highly correlated, but nonetheless, they represented two separate factors. After controlling for demographic factors and country-level variables, both PERMA Well-being and MHS-R were negative predictors of psychological distress. Mature happiness was a better predictor of stress, anxiety, and general distress, while PERMA showed a higher prediction of depression. Mature happiness moderated the relation between the perceived noxious effects of the pandemic and all markers of distress (depression, anxiety, stress, and total DASS-21). Instead, PERMA acted as a moderator in the case of depression and stress. These findings indicate that inner harmony, according to the mature happiness theory, is an essential facet of well-being to be taken into consideration. The results of this study can also orient policies aimed to alleviate the negative effects of the pandemic on mental health through the promotion of well-being.

## Introduction

The term “subjective well-being” was initially introduced by Diener and Emmons (1984), who understood it as a combination of long-term levels of positive affect, lack of negative affect, and satisfaction with life. Diener stated that this construct presents three hallmarks: (1) it is subjective, that is, it depends on the individual experience, (2) it is not the mere absence of negative affect, but also includes the measure of positive states, and (3) it includes a global assessment of one's life rather than a specific domain. Since this initial concept, other approaches to well-being have been provided. For instance, Ryff (1989) proposed the construct of “psychological well-being” as a composite of six domains (self-acceptance, positive relations with others, autonomy, environmental mastery, purpose in life, and personal growth), a model that has received extensive empirical support (Ryff, 2014; Ryff et al., 2016). In a similar vein, Keyes (2002) divided well-being and mental health into three major categories: emotional well-being (described as the presence of positive feelings and absence of negative feelings about life), psychological well-being (private and personal evaluations of one's positive functioning in life), and social well-being (public and social criteria of people's functioning in life including dimensions such as social coherence, social actualization, social integration, social acceptance, and social contribution).

The concept of well-being, which is generally interchangeable with “happiness,” has gained a special interest in the last two decades, along with the rise of positive psychology (Seligman and Csikszentmihalyi, 2000; Seligman, 2002). Happiness has been linked to the experience of positive emotions, positive relationships, engagement, wellness, and meaning in life, among others (Seligman, 2002, 2011). One of the recently extended approaches to happiness and well-being has been provided by Seligman (2011). Seligman's conceptualization of happiness and flourishing is based on five pillars: Positive emotions, Engagement, Relationships, Meaning in life, and Achievement (under the acronym “PERMA,” Seligman, 2011). This model integrates three types of happiness described by Wong (2011): hedonic happiness (having a high positive affect and low negative affect), prudential happiness (being engaged in life), and eudaimonic happiness (the pursuit of virtue and meaning in life and an accompanying sense of fulfillment). The PERMA model has been proved to be useful in the prediction of positive functioning and mental health (e.g., Kern et al., 2015; Butler and Kern, 2016; Giangrasso, 2018). Also, some of its components such as positive emotions and meaning independently have demonstrated to play an important role in clinical areas such as resilience (Fredrickson et al., 2003; Fredrickson, 2009; Wong, 2012; Hicks and Routledge, 2013; Batthyany and Russo-Netzer, 2014).

Beyond its contribution to the understanding and prediction of well-being, the PERMA model is open to the inclusion of new elements into the happiness equation, as suggested by Seligman (2018). For instance, the PERMA approach is built under the assumption that higher levels of the five components (positive emotions, engagement, relationships, meaning, and achievement) act as a buffer against negative emotions and distress, which has been to a certain extent empirically supported (e.g., Butler and Kern, 2016; Umucu et al., 2020). However, this model does not explicitly include the management of negative emotions in its conceptualization of well-being. For instance, PERMA does not mention what type of relationship with unpleasant emotions and thoughts is needed to sustain well-being in times of adversity. This exclusion of negative emotions is comprehensible since the positive psychology movement emerged as an alternative paradigm against mainstream psychology from several decades ago (Seligman, 1998; Seligman and Csikszentmihalyi, 2000), which was strongly focused on psychopathology. For that reason, theoretical propositions from positive psychology have been focused on positivity, reducing the attention to the negative aspects of living. Another potential reason for this overlook of negative emotions is that PERMA, just like the first movement of positive psychology, has been developed and tested in times of global peace and prosperity. However, times have drastically changed with the COVID-19 spread and the incorporation of new elements to the understanding of happiness could significantly contribute to the prediction and promotion of mental health during the current pandemic.

The unprecedented pandemic has altered our lifestyle, generating a new scenario in which social isolation and psychological distress due to confinement, social distancing, fear of getting infected, and economic uncertainty, among others, are globally experienced (e.g., Kirzinger et al., 2020; Qiu et al., 2020; Witters and Harter, 2020). In this context, a model of well-being and happiness that disregards the complex role of negative emotions may be limited. Many people who are used to experiencing high levels of well-being (according to PERMA) can be simultaneously suffering to a great extent from the present collective crisis. For example, let us think of a person who presents high levels of PERMA components, that is, they often experience positive emotions, are highly engaged in what they do, have positive relationships, have found meaning in their life, and have achieved worthy life goals. Although these can be important factors that contribute to their well-being, it is not difficult to imagine that this person can still suffer from serious inner conflicts and distress because of confinement, trauma, worry about the future, and fear of contagion. We believe that this may be just one potential example out of millions worldwide. However, we have found no previous study exploring the boundaries of this model of well-being during difficult situations and proposing a conceptualization of happiness based on an integration of positive and negative aspects of living.

In its initial conceptualizations, subjective well-being included the measure of negative affect Diener and Emmons (1984). For instance, Bradburn (1969) proposed that happiness is a judgment of people about the ratio between their positive affect and their negative affect. That is, a person has more well-being when they experience higher levels of pleasant emotions and lower levels of unpleasant emotions. However, the role of negative affect in well-being and its independence with positive affect have been controversial (Zevon and Tellegen, 1982; Diener and Emmons, 1984; Watson, 1988). For example, Diener and Iran-Nejad (1986) found that at low levels of both, negative and positive types of affect can co-exist, while at higher levels they rarely appear together.

An alternative perspective of well-being is proposed by existential positive psychology (PP2.0, Wong, 2009, 2011), also known as the second wave of positive psychology (Ivtzan et al., 2015; Lomas and Ivtzan, 2016). Existential positive psychology highlights the importance of negative emotions and stressful events in the development of well-being. According to this paradigm, negative aspects of living, such as suffering, trauma, loss, or isolation, although generally considered undesirable, can also serve as promoters of personal growth and resilience (Wong, 2011). Negative emotions have had an adaptive function in human evolution, particularly in terms of protection (Nesse, 2019). In this line, some authors have highlighted the upsides of negative emotions and negative experiences (Calhoun and Tedeschi, 2006; Kashdan and Biswas-Diener, 2014; Ivtzan et al., 2015). However, the adaptive role of negative emotions depends to a great extent on how one relates to them. In the last years, increasing empirical evidence has suggested that the acceptance of negative emotions and thoughts is necessary for optimal functioning (e.g., Levin et al., 2012; Stockton et al., 2019), while rigid avoidance of negative emotions is associated with different forms of psychopathology (Chawla and Ostafin, 2007; Spinhoven et al., 2014). Overall, these studies reflect that, as well as well-being does not simply represent a lack of negative emotions, neither can negative emotions be treated merely as a lack of positive affect.

In line with existential positive psychology, a different conceptualization of well-being called “mature happiness” has been recently proposed (Wong and Bowers, 2018). Mature happiness is defined as a positive state of mind characterized by calmness, acceptance, contentment, inner harmony, and life satisfaction (Wong and Bowers, 2018). This deeply rooted happiness is based on the attunement with all parts of oneself as a whole, including strengths and weaknesses, pleasant and unpleasant emotions. It appeals to a balanced life in which one is at peace with oneself, others, and the world (Haybron, 2013; Wong, 2014). This conceptualization of well-being is related to both subjective and eudaimonic well-being. Just like subjective well-being, mature happiness refers to the feelings of a person and represents an experiential component of well-being. However, the harmony feelings included in mature happiness, in contrast to the positive emotions typically associated with subjective well-being (e.g., joy, satisfaction, excitement, and enthusiasm), are more representative of the mental state resulting from pursuing an eudaimonic life [for a further discussion on the integration of subjective and eudaimonic components of well-being, see Martela and Sheldon (2019)]. In fact, components such as self-acceptance and serenity have been previously linked to eudaimonic well-being (Ryff, 1989; Blasi et al., 2013), while emotional stability has been identified as one of the main features of flourishing (Huppert and So, 2013). Additionally, mature happiness encompasses the *chaironic* type of happiness, which represents feeling blessed or fortunate because of a sense of awe, gratitude, and oneness with the world (Wong, 2011). The adjective “mature” indicates that the development of this harmony-based happiness requires a significant level of personal maturity and self-discipline. Mature happiness captures the notion of balance and harmony from the principles of Confucianism, Dao/Taoism, and Buddhism, particularly from Yin-Yang dialectics (Wong, 2016). Furthermore, this conceptualization of happiness is strongly related to the stoic concept of *apatheia*, which is considered to be a crucial constituent of the eudaimonic life (Pigliucci, 2020). In stoicism, *apatheia* is construed as freedom from disturbing emotions (e.g., distress, fear, lust, or excessive delight) and equanimity in the face of what the world throws us (Pigliucci, 2020).

According to its definition, mature happiness represents a *positive* state of mind. This means that although this type of happiness incorporates an adaptive relationship with negative emotions and personal burdens (e.g., through acceptance, letting it go, and the maintenance of calm whatever comes), it refers to the positive result of living in harmony between both positive and negative aspects of one's life as a whole. This positive state differs from that evaluated by the PERMA-Profiler and traditional measures of subjective well-being (e.g., Diener et al., 1985; Watson et al., 1988). Although instruments such as the PERMA-Profiler (Butler and Kern, 2016) includes a separate subscale of negative affect, the measurement and conceptualization of PERMA themselves do not. Similarly, traditional measures of subjective well-being such as the Positive and Negative Affect Schedule [PANAS, Watson et al., 1988, see also Bradburn (1969)] include the evaluation of negative affect, but as a separate construct of positive affect. However, these measures often fail to evaluate the complexity of emotions (Diener, 1994) and do not adopt a dialectical perspective providing an evaluation of the overall mental state resulting from living in an optimal balance between both positives and negatives.

The conceptualization of happiness as inner harmony has recently gained empirical support. For example, Delle Fave et al. (2016) carried out a cross-cultural study to evaluate laypeople's definitions of happiness. These authors found that inner harmony and relational connectedness were largely the most mentioned facets when people define happiness. The mature happiness conceptualization challenges traditional approaches to well-being and it can be more appropriate for those who are suffering, as it highlights the capacity to maintain well-being despite the negative aspects of one's existence. Thus, mature happiness puts emphasis on the importance of tolerating and embracing uncertainty and suffering to maintain composure in adversity. Besides this focus on endurance, mature happiness is also based on the use of people's inner resources versus external resources, and the seeking of peace instead of excitement, which can be more adaptive and sustained amid a restrictive collective crisis such as the current coronavirus pandemic.

The above-mentioned reasons lead us to consider that inner harmony, according to the mature happiness theory, can be an essential facet of well-being, particularly during difficult times (Dambrun and Ricard, 2011; Delle Fave et al., 2016; Wong and Bowers, 2018). According to this recent theory of happiness and well-being, suffering can be transformed into growth with meaning, mindfulness, and equanimity (Wong, 2020). These elements have been associated with a better adjustment to adverse situations (e.g., Kashdan and Rottenberg, 2010; Boyd et al., 2018; Vos and Vitali, 2018). However, to date, no previous study has analyzed the contribution of mature happiness to the prediction of mental health in adverse situations in comparison with other models such as PERMA.

The present study aimed to analyze the role of mature happiness and PERMA in the prediction of psychological distress during the 1st months of the COVID-19 pandemic. A total of 12,203 participants from 30 countries representing all continents responded to an online survey including the Depression Anxiety Stress Scale (Lovibond and Lovibond, 1995), the PERMA-Profiler (Butler and Kern, 2016), and a revised version of the Mature Happiness Scale [original by Wong and Bowers (2018)] that we have recently validated (not published yet). The hypotheses of the study were the following:

H1: Mature happiness will be moderately associated with PERMA and its different elements (positive emotions, engagement, relationships, meaning, and achievement), but it will not measure the same phenomenon as PERMA.
H2: Both models of well-being will be negatively associated with psychological distress. However, mature happiness will predict psychological distress above and beyond PERMA.
H3: At higher effects of the pandemic on the psychological state, mature happiness will increase its prediction of distress more strongly than PERMA.

## Method

### Participants

The final sample consisted of 12,203 people from 30 countries from all continents. The majority of the participants were female (71.3%) and the average age was 35.52 years (*SD* = 13.21; range 18–85). The inclusion criteria were to be over 18 years old and fluent in any of the languages into which the survey was translated.

### Measures

#### Demographic Variables and Effect of COVID-19 Pandemic

Gender, age, and psychological disorder diagnosis (yes/no) were measured together with the perceived noxious psychological effect of the COVID-19 pandemic (“This pandemic deteriorates my psychological health”). This item was measured on a Likert scale of 1 (“I do not agree at all”) to 7 (“I completely agree”).

#### PERMA-Profiler

The PERMA-Profiler (Butler and Kern, 2016), which measures five aspects of flourishing (positive emotion, engagement, relationships, meaning, and accomplishment with three items each and happiness with one item), was also administered. The composite score of these domains represents PERMA Well-being. In this study, we did not use the PERMA subscales of physical health, loneliness, and negative emotion. Agreement ratings with each item were measured on a Likert scale of 0–6 to be consistent with the questionnaire package [these types of modifications do not affect the performance of questionnaires; see Dawes (2008)]. The internal consistency of the instrument was good in all countries (see **Table 2**).

#### Mature Happiness Scale-Revised

We employed the Mature Happiness Scale-Revised [MHS-R, original by Wong and Bowers (2018)], which measures inner harmony through nine items (e.g., “I am able to maintain inner peace,” “I have learned to let go of all my cares and burdens”). Respondents rated each of these items on a Likert scale from 1 (“not at all”) to 5 (“all of the time”), with a total score of 40. Cronbach's alphas were good in all participating countries (see **Table 2**). This revised version has been validated in different languages and can be shared upon request to the authors.

#### Depression Anxiety Stress Scale

To measure psychological distress, we used the Depression Anxiety Stress Scale (DASS-21; Lovibond and Lovibond, 1995). This questionnaire contains 21 items describing negative emotional states during the last week. Responses are collected on a 4-point Likert-type scale ranging from 0 (“did not apply to me at all”) to 3 (“applied to me very much, or most of the time”). The scale is composed of three subscales (depression, anxiety, and stress) that form one global score of psychological distress. Internal consistency of the measure was adequate in all participating countries (see **Table 2**).

### Procedure

The present study was part of a large-scale international project. We recruited the participants through snowball sampling using social media announcements and invitations by email. A list of 43 international collaborators (experienced researchers in psychology) from the participating countries shared a link to the online survey with their acquaintances and asked them to share it with their respective contacts, therefore creating a chain of messages. We asked collaborators to collect the majority of the responses from people who were not students or psychologists in order to have a more representative sample of the general population. Respondents did not receive any incentives for their participation, with the exception of a part of the Canadian sample that was recruited *via* MTurk. Participants responded to questions about demographic data, psychological effects of COVID-19, and completed the questionnaires of PERMA and MHS-R. Upon completion of the questionnaire, they were fully debriefed. To adapt the survey to each language, already validated versions of the instruments were used. If an instrument had not been previously validated in a specific language, the validation process was initiated by the international collaborators implementing the best practices [see Beaton et al. (2000)]. These adapted versions and their psychometric properties can be shared upon request. The study was approved by multiple ethics committees from the participating countries.

### Data Analytic Strategy

Data were assessed with MPlus (Version 8.4; Muthén and Muthén, 2016) and SPSS (Version 25). Data of participants who did not fill in 50% or more of any of the questionnaires or showed straightlining were removed. As a very low percentage of participants with COVID-19 diagnosis was observed (below 1%), and these people most probably had a significantly different experience (e.g., being more distressed or hospitalized) during the 1st months of the pandemic, when significant information about the virus was lacking. These data were also removed. We did so to maintain the homogeneity of the sample, as the addition of a variable with an extremely imbalanced class distribution could have caused the predictive model to be biased and inaccurate. Missing data on the questionnaires (<0.1%) was missing completely at random, thus missing values were replaced with the expectation maximization algorithm. There were some missing data on the demographic variables, specifically; participant's age was not measured in 136 cases due to an error in the US sample. These values were replaced with the country average.

Confirmatory factor analyses (CFAs) on item level were conducted to assess the relationships between the PERMA-Profiler and the MHS-R. We evaluated a one-factor solution (all 23 items of PERMA-Profiler and MHS-R load on a single general factor of well-being), a two-factor solution (PERMA items load on PERMA, MHS-R items load on MHS-R, and the two correlate), and an alternative two-factor solution (MHS-R items together with happiness and positive emotion load on MHS-R, the rest of the PERMA items load on PERMA, and the two factors correlate). Analyses were conducted using the Satorra-Bentler correction and solutions were assessed using conventional fit indices, as adjusted χ^2^ values are sensitive to sample size. The Comparative Fit Index (CFI), the Tucker–Lewis index (TLI), the Root-Mean-Square Error of Approximation (RMSEA), and the Standardized Root-Mean-Square Residual (SRMR) were evaluated that typically have the following cutoff scores: RMSEA and SRMR ≤ 0.080; CFI and TLI ≥ 0.90 (e.g., Browne and Cudeck, 1993; Hu and Bentler, 1998). As the aim was to compare different models and determine the relationship between PERMA and MHS-R and not covariance matrix hypothesis testing, the aforementioned cutoff scores were not used as strict decision rules (Marsh et al., 2004). Nested models (one-factor model vs. two-factor models) were compared with the Satorra-Bentler scaled χ2 difference tests and non-nested models (two-factor models) were contrasted by assessing fit indices and AIC (Akaike Information Criterion) difference (Burnham and Anderson, 2004).

Invariance (configural, measurement and scalar) was assessed across the 30 participating countries. Successively restrictive models were compared with the Satorra-Bentler scaled χ^2^ difference tests and with CFI, RMSEA, and SRMR difference scores. For large sample sizes as the present one, traditional cutoff scores of ≤ −0.010 for CFI, ≤ 0.015 for RMSEA and ≤ 0.030 / ≤ 0.010 for SRMR (it depends on the level of testing) show invariance (Chen, 2007).

Relationships between variables were assessed with Pearson's correlation coefficient between continuous variables and between continuous and dichotomous variables, and with χ^2^ tests between two dichotomous variables.

Multilevel modeling (MLM) analyses were applied in order to compare the predictive power of perceived psychological effects of the pandemic and their respective interactions with PERMA Well-being and MHS-R on depression, stress, anxiety, and DASS-21 total scores. Baseline models with random intercepts were compared with nested models using χ^2^ difference tests. Interactions were further evaluated with eight separate simple slope analyses (PROCESS macro; Hayes, 2017) at high (+1 SD), medium, and low levels (−1 SD) of the moderator variables (PERMA Well-being and MHS-R). Standardized variables were used in all analyses.

## Results

### Descriptive Statistics

Demographic data of the participants, mean scores, and standard deviations can be observed in Tables 1, 2. Of note, only 17.7% of participants were students and 8.7% psychology professionals. Both PERMA Well-being and MHS-R were slightly negatively skewed, showing that participants generally reported relatively high levels of well-being (PERMA Well-being: kurtosis = 0.82, skewness = −0.99; MHS-R: kurtosis = −0.07, skewness = −0.42).

**Table 1:** Socio-demographic characteristics of the sample and markers of psychological problems.

**Country**	***n***	**Gender**	**Age**		**Psychological diagnosis**	**Psychological effect of COVID-19**	**Depression**	**Anxiety**	**Stress**	**DASS-Total**
		**F%**	***M* (*SD*)**	**Range**	**yes %**	***M (SD)***	***M (SD)***	***M (SD)***	***M (SD)***	***M (SD)***
Algeria	264	67.9	32.58 (10.35)	18-69	4.2	3.36 (2.20)	9.74 (9.70)	8.20 (9.00)	11.99 (10.63)	29.94 (27.25)
Argentina	163	74.8	37.50 (11.67)	18-70	11.0	3.86 (2.06)	8.88 (9.47)	7.45 (8.35)	12.52 (10.01)	28.85 (25.27)
Australia	63	85.7	44.19 (10.65)	18-73	15.9	3.71 (1.88)	8.19 (8.13)	5.46 (6.39)	13.56 (9.65)	27.21 (20.87)
Bangladesh	344	39.8	25.35 (7.41)	18-78	0	4.12 (2.17)	13.30 (9.24)	12.63 (9.14)	15.01 (9.61)	40.94 (25.87)
Brazil	386	75.6	37.94 (12.71)	18-77	22.8	4.86 (1.91)	12.13 (10.47)	6.61 (7.68)	12.89 (10.21)	31.63 (25.76)
Canada	394	52.4	36.80 (13.23)	18-84	15.7	4.26 (1.87)	11.36 (10.34)	6.75 (7.61)	13.18 (9.95)	31.28 (25.02)
Colombia	130	88.5	39.54 (12.27)	18-70	11.5	3.94 (2.08)	10.92 (10.50)	8.38 (9.89)	15.58 (11.12)	34.89 (28.46)
Egypt	293	71.0	37.23 (11.50)	18-84	2.7	3.48 (1.77)	10.62 (9.68)	7.45 (8.13)	12.31 (10.03)	30.38 (25.18)
France	442	78.7	46.60 (11.65)	18-81	3.6	3.35 (1.90	10.01 (9.79)	5.06 (6.61)	12.89 (10.56)	28.00 (23.51)
Germany	296	69.3	40.78 (15.04)	18-79	5.1	2.95 (1.85)	5.99 (7.73)	2.71 (5.02)	8.78 (8.86)	17.48 (18.67)
Hungary	282	89.3	37.36 (12.40)	18-71	7.1	4.22 (1.92)	9.22 (9.73)	7.10 (7.95)	15.03 (9.89)	31.35 (23.83)
India	602	56.7	25.75 (7.94)	18-85	1.0	2.68 (1.90)	6.41 (7.53)	6.19 (7.06)	6.90 (7.60)	19.50 (20.41)
Indonesia	289	73.0	24.78 (9.46)	18-59	4.2	3.62 (1.99)	7.74 (7.76)	7.47 (7.40)	12.53 (9.18)	27.74 (22.26)
Italy	536	75.9	34.50 (14.67)	18-80	4.7	4.00 (1.94)	10.98 (7.91)	5.62 (6.34)	13.24 (8.31)	29.84 (19.20)
Lebanon	329	65.3	28.34 (11.59)	18-69	10.6	4.19 (2.07)	13.62 (11.32)	9.57 (9.55)	15.74 (10.54)	38.93 (28.32)
Mexico	717	80.3	40.76 (13.34)	18-80	9.6	3.60 (2.10)	8.26 (9.01)	6.60 (8.28)	13.55 (9.82)	28.42 (24.72)
New Zealand	73	80.8	44.89 (11.30)	20-74	41.1	4.03 (1.97)	11.51 (10.55)	7.34 (9.01)	15.23 (9.66)	34.08 (25.49)
Nigeria	435	31.5	33.34 (8.67)	19-64	0	3.10 (2.01)	5.53 (7.49)	5.00 (7.24)	6.57 (8.04)	17.10 (21.23)
Pakistan	426	61.3	28.59 (10.33)	18-80	1.4	3.42 (2.03)	11.54 (9.31)	10.02 (8.79)	13.37 (9.22)	34.93 (25.53)
Poland	332	81.6	32.69 (12.18)	18-82	17.2	4.66 (1.92)	12.59 (11.42)	8.90 (9.45)	17.36 (11.08)	38.85 (28.83)
Portugal	522	72.4	38.93 (12.20)	18-75	6.9	4.23 (1.85)	7.49 (7.49)	5.26 (7.20)	13.36 (9.88)	26.11 (22.06)
Romania	557	70.7	32.78 (11.59)	18-69	2.0	3.18 (1.84)	8.30 (8.54)	6.02 (7.73)	11.16 (9.66)	25.48 (23.16)
Russia	324	89.8	44.38 (11.03)	19-79	5.2	3.79 (1.99)	9.19 (8.41)	6.23 (6.96)	14.17 (9.62)	29.59 (21.72)
Slovenia	1345	83.2	34.39 (13.67)	18-81	5.5	3.86 (2.05)	10.39 (10.34)	5.85 (7.60)	14.08 (10.74)	30.33 (25.96)
Spain	723	77.0	36.51 (11.81)	18-73	11.5	3.86 (2.03)	9.04 (9.51)	6.47 (7.66)	13.04 (9.29)	28.55 (23.79)
Sweden	314	84.3	41.05 (12.23)	20-75	11.5	3.86 (1.95)	9.60 (8.61)	3.79 (6.46)	11.34 (8.57)	24.73 (19.61)
Thailand	422	35.1	34.23 (10.83)	18-70	4.0	3.51 (1.75)	5.54 (7.17)	4.92 (6.24)	8.96 (8.17)	19.42 (20.20)
Turkey	322	60.9	27.27 (8.59)	18-61	6.2	4.19 (2.11)	12.97 (10.36)	8.55 (8.00)	14.35 (10.23)	35.87 (25.86)
United Kingdom	514	88.1	42.33 (15.22)	18-76	25.7	4.47 (1.92)	15.06 (11.80)	10.66 (11.04)	18.42 (11.75)	44.14 (31.58)
United States	364	78.6	44.37 (12.33)	18-77	23.1	4.18 (2.01)	11.56 (11.09)	6.81 (8.14)	13.93 (10.99)	32.30 (27.23)
Total	12.203	71.3	35.52 (13.21)	18-85	8.3	3.79 (2.03)	9.81 (9.70)	6.80 (8.07)	12.90 (10.15)	29.52 (25.18)

**Table 2:** Descriptive statistics of Mature Happiness and PERMA subscales (*N* = 12,203).

**Country**	**Mature happiness**	**Positive emotion**	**Engagement**	**Relationships**	**Meaning**	**Accomplish.**	**Happiness**	**PERMA well-being**
	***M* (*SD*)**	***M* (*SD*)**	***M* (*SD*)**	***M* (*SD*)**	***M* (*SD*)**	***M* (*SD*)**	***M* (*SD*)**	***M* (*SD*)**
Algeria	26.72 (5.24)	3.93 (1.42)	3.81 (1.26)	3.80 (1.54)	4.15 (1.51)	3.91 (1.33)	3.94 (1.68)	3.92 (1.20)
Argentina	25.96 (5.39)	4.03 (1.32)	4.24 (1.15)	4.33 (1.39)	4.54 (1.44)	3.94 (1.27)	4.17 (1.40)	4.21 (1.12)
Australia	25.40 (4.01)	4.21 (1.06)	4.41 (1.07)	4.69 (1.18)	4.83 (1.30)	4.26 (1.09)	4.56 (0.93)	4.48 (0.90)
Bangladesh	24.71 (6.22)	3.35 (1.51)	2.83 (1.23)	3.45 (1.57)	3.55 (1.55)	3.32 (1.49)	3.50 (1.81)	3.31 (1.30)
Brazil	23.36 (5.72)	3.77 (1.30)	4.14 (1.11)	4.24 (1.18)	4.12 (1.30)	3.82 (1.30)	3.98 (1.31)	4.01 (1.06)
Canada	24.54 (5.36)	3.79 (1.22)	4.01 (1.12)	4.15 (1.26)	4.15 (1.31)	3.94 (1.05)	4.09 (1.24)	4.01 (1.01)
Colombia	24.71 (5.70)	3.96 (1.26)	4.32 (1.01)	4.46 (1.25)	4.66 (1.18)	4.00 (1.15)	4.31 (1.25)	4.28 (0.89)
Egypt	24.51 (4.79)	4.04 (1.26)	4.00 (1.18)	4.19 (1.40)	4.33 (1.51)	4.01 (1.36)	4.75 (1.43)	4.15 (1.14)
France	23.34 (3.46)	4.18 (0.98)	4.45 (0.92)	4.31 (1.11)	4.22 (1.20)	4.35 (0.91)	4.35 (1.12)	4.31 (0.82)
Germany	25.62 (4.24)	4.28 (0.94)	4.17 (0.97)	4.72 (1.02)	4.62 (0.95)	4.14 (0.86)	4.57 (1.00)	4.40 (0.73)
Hungary	23.76 (5.14)	3.98 (1.11)	4.30 (0.96)	4.59 (1.32)	4.59 (1.21)	4.38 (0.96)	4.15 (1.29)	4.36 (0.87)
India	26.59 (5.85)	4.01 (1.38)	3.77 (1.29)	4.15 (1.50)	4.24 (1.44	3.99 (1.43)	4.27 (1.52)	4.05 (1.23)
Indonesia	27.63 (4.59)	4.47 (1.05)	4.63 (0.87)	4.58 (1.11)	4.64 (1.05)	4.35 (0.97)	4.60 (1.28)	4.54 (0.85)
Italy	23.01 (5.14)	3.67 (1.16)	4.33 (1.04)	4.26 (1.16)	4.20 (1.25)	3.90 (1.08)	4.08 (1.15)	4.07 (0.92)
Lebanon	24.83 (5.61)	3.82 (1.31)	4.31 (1.19)	4.07 (1.35)	4.15(1.42)	4.06 (1.23)	3.95 (1.34)	4.07 (1.09)
Mexico	27.26 (4.90)	4.43 (1.05)	4.69 (0.94)	4.84 (1.06)	4.86 (1.06)	4.25 (1.10)	4.77 (1.04)	4.62 (0.87)
New Zealand	22.68 (5.11)	3.64 (1.36)	3.90 (1.26)	4.13 (1.51)	4.09 (1.39)	3.80 (1.11)	3.82 (1.49)	3.91 (1.17)
Nigeria	28.35 (5.44)	4.43 (1.27)	3.88 (1.24)	4.20 (1.29)	4.65 (1.27)	4.18 (1.27)	4.45 (1.28)	4.28 (1.12)
Pakistan	25.04 (6.37)	3.58 (1.52)	3.68 (1.40)	3.77 (1.44)	3.78 (1.32)	3.46 (1.48)	3.94 (1.73)	3.67 (1.32)
Poland	23.39 (5.31)	3.75 (1.19)	4.28 (1.10)	4.20 (1.32)	3.99 (1.51)	3.88 (1.13)	4.00 (1.26)	4.02 (1.03)
Portugal	26.26 (4.84)	4.19 (1.08)	4.32 (1.01)	4.59 (1.16)	4.61 (1.20)	4.12 (0.99)	4.60 (1.12)	4.38 (0.92)
Romania	27.18 (5.31)	4.27 (1.13)	4.48 (1.05)	4.61 (1.15)	4.74 (1.19)	4.31 (1.08)	4.28 (1.22)	4.47 (0.94)
Russia	24.48 (4.84)	3.90 (1.03)	4.41 (0.91)	4.35 (1.11)	4.50 (0.99)	4.33(0.81)	4.31 (1.07)	4.30 (0.74)
Slovenia	24.47 (5.06)	4.28 (1.08)	4.40 (0.97)	4.43 (1.20)	4.50(1.29)	4.28 (0.98)	4.35 (1.25)	4.38 (0.92)
Spain	24.56 (5.15)	3.97 (1.21)	4.29 (1.14)	4.54 (1.21)	4.39 (1.38)	3.79 (1.25)	4.22 (1.21)	4.20 (1.07)
Sweden	24.18 (5.08)	4.06 (1.04)	3.99 (1.05)	4.54 (1.13)	4.48 (1.25)	4.04 (1.02)	4.04 (1.16)	4.21 (. 89)
Thailand	26.74 (4.79)	4.19 (1.02)	4.24 (1.01)	4.27 (1.06)	4.41 (1.11)	4.36 (1.06)	4.30 (1.18)	4.30 (0.91)
Turkey	25.39 (5.54)	4.01 (1.18)	4.30 (1.10)	4.10 (1.22)	4.16 (1.16)	4.09 (1.01)	4.19 (1.32)	4.13 (0.97)
United Kingdom	22.71 (5.57)	3.51 (1.33)	3.88 (1.34)	4.07 (1.45)	3.92 (1.50)	3.68 (1.29)	3.82 (1.45)	3.81 (1.18)
United States	24.60 (5.46)	3.87 (1.11)	4.20 (1.13)	4.40 (1.22)	4.51 (1.17)	4.09 (1.19)	4.24 (1.22)	4.22 (0.95)
Total	25.13 (5.43)	4.02 (1.22)	4.19 (1.15)	4.32 (1.28)	4.36 (1.32)	4.05 (1.18)	4.25 (1.32)	4.19 (1.05)

Significant differences between countries were observed on all variables, such as age, *F*_(29)_ = 88.18, *p* < 0.001, η_*p*_*2* = 0.174, gender, Cramer's *V* = 0.331, *p* < 0.001, psychological diagnosis, Cramer's *V* = 0.265, *p* < 0.001, and psychological effect of the COVID-19 pandemic, *F*_(29)_ = 27.01, *p* < 0.001, η_*p*_*2* = 0.060, PERMA Well-being, *F*_(29)_ = 28.92, *p* < 0.001, η_*p*_*2* = 0.064, and MHS-R, *F*_(29)_ = 34.82, *p* < 0.001, η_*p*_*2* = 0.077, warranting the inclusion of all these variables in the analyses.

### Measurement Models Including PERMA Well-Being and MHS-R

**Figure 1:**
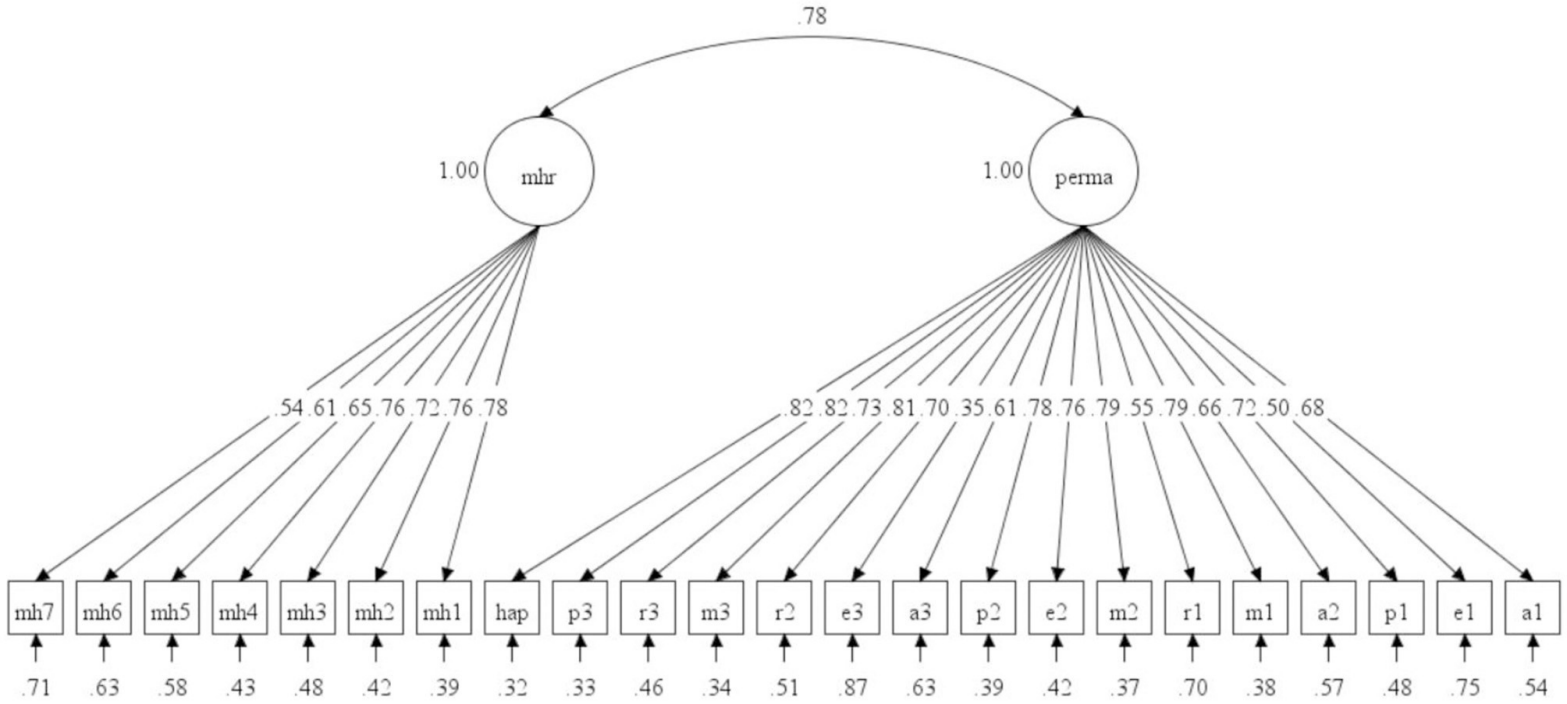
Standardized solution for the two-factor model of PERMA and Mature Happiness. Error is not shown but it was specified for each variable. Error covariances were not allowed (*N* = 12,203).

The one-factor solution (all 23 items loading on a general well-being factor) showed an unsatisfactory model fit χ^2^ = 20348.41, *df* = 230, *p* < 0.001, CFI = 0.828, TLI = 0.811, RMSEA = 0.085 [90% CI 0.084, 0.086], SRMR = 0.063. Standardized factor loadings ranged between 0.35 and 0.86. The two-factor solution showed the following fit: χ^2^ = 13820.80, *df* = 229, *p* < 0.001, CFI = 0.884, TLI = 0.872, RMSEA = 0.070 [90% CI 0.069, 0.071], SRMR = 0.050. Standardized factor loadings ranged between 0.36 and 0.83 for PERMA and between 0.54 and 0.78 for MHS-R. The latent correlation between PERMA and MHS-R was 0.78. The robust χ^2^ difference test showed that the two-factor solution was superior to the one-factor solution, Δχ^2^ (1) = 6329.72, *p* < 0.001. For this solution, see Figure 1.

We also tested an alternative two-factor solution with positive emotion and happiness loading on MHS-R, χ^2^ = 16885.79, *df* = 229, *p* < 0.001, CFI = 0.858, TLI = 0.843, RMSEA = 0.077 [90% CI 0.076, 0.078], SRMR =0.057. In this case, standardized factor loadings ranged between 0.36 and 0.82 for PERMA and between 0.47 and 0.83 for MHS-R. The latent correlation between the two factors was 0.89. This model was significantly better than the one-factor model, Δχ^2^ (1) = 3516.96, *p* < 0.001, however, it was worse than the original two-factor model, as evidenced by differences in fit indices and ΔAIC = 4451.56 (model including PERMA and MHS-R as separate factors: AIC = 804925.66; model including happiness and positive emotions in MHS-R: AIC = 809377.23).

Configural, χ^2^ = 27271.60, *df* = 6,870, *p* < 0.001, CFI = 0.853, TLI = 0.838, RMSEA = 0.085 [90% CI 0.084, 0.087], SRMR = 0.061, metric, χ^2^ = 30431.78, *df* = 7,479, *p* < 0.001, CFI = 0.835, TLI = 0.832, RMSEA = 0.087 [90% CI 0.086, 0.088], SRMR = 0.100, and scalar, χ^2^ = 38841.00, *df* = 8,088, *p* < 0.001, CFI = 0.778, TLI = 0.792, RMSEA = 0.097 [90% CI 0.096, 0.098], SRMR = 0.114, invariance was assessed and the results showed impaired invariance.

### Relationships Between Variables

Results showed relations between variables in the theoretically expected directions, depicted in Table 3. For instance, older participants and males tended to report higher levels of MHS-R and PERMA Well-being, and both of the aforementioned measures were negatively related to markers of psychological distress. People who reported psychological disorder diagnosis tended to show lower levels of MHS-R and PERMA Well-being and higher levels of depression, anxiety, and stress.

**Table 3:** Relationships between of the main variables of the study (*N* = 12,203).

	**1.**	**2.**	**3.**	**4.**	**5.**	**6.**	**7.**	**8.**	**9.**	**10.**	**11.**	**12.**	**13.**	**14.**	**15.**
1. Depression															
2. Anxiety	0.673^***^														
3. Stress	0.753^***^	0.721^***^													
4. DASS-21 Total	0.904^***^	0.870^***^	0.924^***^												
5. MHS-R	−0.487^***^	−0.353^***^	−0.454^***^	−0.484^***^											
6. Positive emotion	−0.541^***^	−0.358^***^	−0.397^***^	−0.483^***^	0.698^***^										
7. Engagement	−0.310^***^	−0.204^***^	−0.159^***^	−0.249^***^	0.443^***^	0.624^***^									
8. Relationship	−0.367^***^	−0.237^***^	−0.224^***^	−0.308^***^	0.509^***^	0.688^***^	0.516^***^								
9. Meaning	−0.469^***^	−0.290^***^	−0.283^***^	−0.388^***^	0.617^***^	0.760^***^	0.635^***^	0.693^***^							
10. Accomplishment	−0.399^***^	−0.265^***^	−0.255^***^	−0.341^***^	0.554^***^	0.689^***^	0.642^***^	0.526^***^	0.712^***^						
11. Happiness	−0.477^***^	−0.311^***^	−0.333^***^	−0.418^***^	0.630^***^	0.792^***^	0.531^***^	0.681^***^	0.698^***^	0.585^***^					
12. PERMA Well-being	−0.500^***^	−0.325^***^	−0.319^***^	−0.425^***^	0.675^***^	0.895^***^	0.790^***^	0.817^***^	0.899^***^	0.831^***^	0.808^***^				
13. COVID-19 effect	0.473^***^	0.386^***^	0.524^***^	0.517^***^	−0.297^***^	−0.253^***^	−0.089^***^	−0.114^***^	−0.170^***^	−0.147^***^	−0.224^***^	−0.189^***^			
14. Age	−0.163^***^	−0.162^***^	−0.136^***^	−0.170^***^	0.110^***^	0.109^***^	0.101^***^	0.058^***^	0.150^***^	0.122^***^	0.092^***^	0.127^***^	−0.099^***^		
15. Gender	−0.100^***^	−0.090^***^	−0.177^***^	−0.139^***^	0.122^***^	0.012	−0.068^***^	−0.076^***^	−0.021^*^	−0.009	−0.007	−0.036^***^	−0.104^***^	−0.042^***^	
16. Psych. diagnosis	0.238^***^	0.224^***^	0.212^***^	0.249^***^	−0.229^***^	−0.207^***^	−0.118^***^	−0.097^***^	−0.164^***^	−0.144^***^	−0.185^***^	−0.176^***^	0.165^***^	0.025^**^	80.99^***^
Cronbach's alpha	0.88	0.83	0.88	0.94	0.86	0.83	0.62	0.79	0.87	0.77	N/A	0.94	N/A	N/A	N/A

* *p < 0.05*;

** *p < 0.01*;

*** *p < 0.001. All p-values are two-tailed*.

*All data represents r, with the exception of the relationship between gender and psychological diagnosis, where χ^2^ is reported*.

MHS-R scores moderately correlated with all subscales of the PERMA-Profiler, ranging from 0.44 (in the case of Engagement) to 0.70 (Positive Emotions). Of note, MHS-R showed higher correlations with anxiety, stress, and previous psychological disorder diagnosis than PERMA Well-being, while the latter seemed to be more strongly related to depression as compared to MHS-R.

### Predictive Power for Depression, Stress, and Anxiety

Four separate MLM analyses were conducted on depression, stress, anxiety, and total DASS-21 scores. Null models (depression:−2LL = 33978.552; anxiety:−2LL = 34029.577; stress:−2LL = 33888.017; DASS-21 total score:−2LL = 33958.677) showed that effect of country was small but significant (depression: Wald *Z* = 3.65, *p* < 0.001, ICC = 0.062; anxiety Wald *Z* = 3.64, *p* < 0.001, ICC = 0.062; stress: Wald *Z* = 3.66, *p* < 0.001, ICC = 0.066; DASS-21 total score: Wald *Z* = 3.67, *p* < 0.001, ICC = 0.064). Nested, full models were significantly better than null models (see Table 4).

**Table 4:** Multilevel modeling predicting markers of psychological problems.

	**Depression**	**Anxiety**	**Stress**	**DASS-21 total**
	***B* (*SE*)**	***B* (*SE*)**	***B* (*SE*)**	***B* (*SE*)**
**Individual-level variables**				
Age	−0.087 (0.007)^***^	−0.061 (0.008)^***^	−0.080 (0.008)^***^	−0.085 (0.007)^***^
Gender (female)	0.085 (0.016)^***^	0.130 (0.18)^***^	0.176 (0.016)^***^	0.146 (0.016)^***^
Psychological diagnosis	−0.268 (0.026)^***^	−0.440 (0.029)^***^	−0.213 (0.027)^***^	−0.330 (0.026)^***^
Psychological effect of COVID-19	0.341 (0.007)^***^	0.295 (0.008)^***^	0.401 (0.007)^***^	0.388 (0.007)^***^
PERMA Well-being	−0.331 (0.010)^***^	−0.099 (0.011)^***^	−0.051 (0.010)^***^	−0.180 (0.010)^***^
Mature Happiness (MHS-R)	−0.107 (0.010)^***^	−0.168 (0.011)^***^	−0.257 (0.010)^***^	−0.198 (0.010)^***^
**Country-level variables**				
Severity of the pandemic	0.027 (0.039)	−0.081 (0.051)	0.033 (0.046)	−0.001 (0.044)
GDP	−0.009 (0.036)	−0.073 (0.046)	−0.028 (0.041)	−0.038 (0.039)
**Interactions**				
Well-being ^X^ Psych. effect COVID-19	−0.060 (0.009)^***^	0.015 (0.010)	−0.033 (0.009)^***^	−0.005 (0.009)
MHS-R ^X^ Psych. effect COVID-19	−0.083 (0.009)^***^	−0.089 (0.010)^***^	−0.098 (0.009)^***^	−0.100 (0.009)^***^
**Covariance parameters**				
Residual variance	0.537 (0.007)^***^	0.704 (0.009)^***^	0.580 (0.007)^***^	0.540 (0.007)^***^
Intercept variance	0.012 (0.005)^**^	0.029 (0.008)^**^	0.024 (0.007)^**^	0.021 (0.006)^**^
−2 log likelihood	27180.858	30493.272	28147.734	28147.734
χ^2^ (*df*) difference with null model (ML)	6686.939(10)^***^	3603.887(10)^***^	5810.058(10) ^***^	6650.364(10)^***^

*All coefficients are standardized*.

* *p < 0.05*;

** *p < 0.01*;

*** *p < 0.001*.

Results indicated that generally, females and younger participants tended to report higher levels of depression, anxiety, stress, and total DASS-21 levels (see Table 4). Participants with psychological disorder diagnosis and those who perceived a higher psychological impact caused by COVID-19 showed higher levels on all dependent variables. Both PERMA Well-being and MHS-R were significant predictors of all markers of psychological distress. In the case of depression, PERMA Well-being was a stronger predictor than as compared to MHS-R, while in the cases of anxiety, stress, and total DASS-21 scores, MHS-R was established as a more prominent predictor than PERMA Well-being. Country-level variables (GDP, severity of the pandemic) seemed not to be related to the dependent variables.

Analyses of interaction terms showed that MHS-R was a significant moderator between the psychological effects of pandemic and all markers of psychological problems assessed in this study (see Table 4). PERMA Well-being showed to be a moderator between psychological effects of pandemic and in the case of depression and stress but not in case of anxiety and total DASS-21 scores. Single slopes revealed that in each case, MHS-R acted as a protective factor, that is, higher levels of MHS-R weakened the relationships between the psychological effects of the pandemic and levels of psychological problems as evidenced by depression, anxiety, stress, and total DASS-21 levels (see Table 5). A similar protective role of PERMA Well-being was observed in the case of depression and stress but this protective role was not significant with anxiety and total DASS-21 scores as dependent variables, although a tendency was observed in that direction.

**Table 5:** Multilevel modeling predicting markers of psychological problems.

**Outcome**	**Level of moderator**	**Predictor**
	*Psychological effect of COVID-19*	*PERMA Well-being*
Depression	Low	−0.293 (0.010)^***^
	Medium	−0.425 (0.007)^***^
	High	−0.559 (0.010)^***^
Anxiety	Low	−0.213 (0.011)^***^
	Medium	−0.261 (0.008)^***^
	High	−0.308 (0.011)^***^
Stress	Low	−0.173 (0.010)^***^
	Medium	−0.228 (0.008)^***^
	High	−0.283 (0.010)^***^
DASS-21 Total	Low	−0.251 (0.010)^***^
	Medium	−0.339 (0.007)^***^
	High	−0.428 (0.010)^***^
	*Psychological effect of COVID-19*	*Mature Happiness*
Depression	Low	−0.239 (0.011)^***^
	Medium	−0.368 (0.008)^***^
	High	−0.498 (0.010)^***^
Anxiety	Low	−0.174 (0.011)^***^
	Medium	−0.254 (0.008)^***^
	High	−0.334 (0.011)^***^
Stress	Low	−0.239 (0.011)^***^
	Medium	−0.320 (0.008)^***^
	High	−0.402 (0.010)^***^
DASS-21 Total	Low	−0.244 (0.010)^***^
	Medium	−0.352 (0.007)^***^
	High	−0.461 (0.011)^***^

* *p < 0.05*;

** *p < 0.01*;

*** *p < 0.001*.

*All variables were standardized. Significant interactions according to the original full models are highlighted*.

As can be seen in Table 5, MHS-R increased its predictive power on all markers of distress (depression, anxiety, stress, and total DASS-21) at higher psychological effects of the pandemic. In comparison with PERMA Well-being, this increase was more pronounced in the case of stress while it was similar in depression. That is, at higher psychological effects of the pandemic, mature happiness becomes a more robust predictor of stress than PERMA.

## Discussion

This study analyzed the role of PERMA and mature happiness, the latter of which being a recent conceptualization of well-being based on inner harmony (Wong and Bowers, 2018), in the prediction of psychological distress during the 1st months of the COVID-19 pandemic. For that purpose, 12,203 people representing 30 countries from all continents participated in an online survey and three hypotheses were tested.

H1 predicted that mature happiness would be a similar but distinct construct as PERMA. The results seemed to confirm this hypothesis. MHS-R scores showed moderate correlations with PERMA Well-being and its different subscales (positive emotions, engagement, meaning in life, achievement, and general happiness), with the exception of the subscale of relationships. In this case, correlations were lower but still significant). According to the CFAs performed, the one-factor solution showed an unsatisfactory model fit. In contrast, the model that best fitted our data was a two-factor solution in which MHS-R and PERMA Well-being appeared as two distinct but correlated factors. The latent correlation between the two factors was 0.78. This strong correlation can be explained by the fact that inner harmony represents a positive state of mind which is related but more complex than the positive emotions included in PERMA (e.g., joy, contentment, and excitement). Also, mature happiness may be conceived as the mental state constituent of a meaningful life (Wong, 2020), which in turn is represented in PERMA. Nonetheless, our findings suggest that although the two models of well-being are associated, they represent different constructs. The PERMA-Profiler has been recently found to measure the same type of well-being as Diener's model of subjective well-being (Goodman et al., 2018). The current results indicate that this was not the case between PERMA and mature happiness. The subsequent analyses provided additional support for this differentiation between both models.

In line with H2, PERMA Well-being and mature happiness were negatively associated with psychological problems (including depression, anxiety, and stress). These findings are consistent with previous studies reporting a protective role of subjective well-being against psychological distress (e.g., Ong et al., 2006; Butler and Kern, 2016; Gloria and Steinhardt, 2016; Pezirkianidis et al., 2019). In the context of the COVID-19 pandemic, a general decrease in happiness has been reported because of the implementations of restrictive measures such as lockdown (e.g., Greyling et al., 2020). However, in comparison with mental distress, subjective well-being seems to be more resilient to change (e.g., Sibley et al., 2020). In agreement with our results, mental well-being and some of its components, such as positive emotions and the presence of meaning in life have been associated with lower levels of distress during the pandemic (Arslan and Yildirim, 2020; Grover et al., 2020; Moroń and Biolik-Moroń, 2021; Paredes et al., 2021). In general, these results support the importance of focusing on subjective and eudaimonic well-being, rather than paying attention only to mental distress, due to its protective role against the noxious effects of the present collective crisis.

When PERMA and mature happiness were compared, mature happiness showed some advantage in the prediction of distress, as expected according to H2. Although the predictive power of both models on general distress was similar, mature happiness was a better predictor of stress and anxiety than PERMA. In contrast, PERMA showed a higher predictive power on depression. However, when the perceived psychological effects of the pandemic were introduced into the equation, the differences between PERMA and mature happiness in the prediction of depression vanished. Indeed, regarding the moderation effect of both models in the relationship between the psychological effects of the pandemic and psychopathological symptoms, mature happiness demonstrated certain superiority. For instance, MHS-R scores moderated the relationship between psychological effects of the pandemic and all markers of distress (depression, stress, anxiety, and general distress) whereas PERMA-Profiler scores moderated this relationship only in the case of depression and stress.

Notably, at higher psychological effects of the pandemic, mature happiness increased its prediction of stress above PERMA, while a similar tendency was observed in the case of depression between both models. Altogether, the latter findings support H3 suggesting that when individuals are more psychologically affected by the conditions of the COVID-19 crisis, mature happiness may play a higher protective role than the PERMA composite [see Wong (2020)]. This superiority, however, was not observed in the case of depressive symptoms. A potential reason may be that PERMA includes a specific measure of meaning in life, which has been shown to be particularly associated with decreased levels of depression (Steger et al., 2006; Disabato et al., 2017; Carreno et al., 2020). Nonetheless, the moderation effects of mature happiness and PERMA were similar in depression, indicating that mature happiness may also be linked to eudaimonic well-being, as previously suggested (Wong, 2011; Wong and Bowers, 2018). On the one hand, mature happiness may be considered as the outcome of living a meaningful life (Wong, 2020). On the other hand, pursuing harmony with oneself, others, and the world in itself can be an important contributor to the experience of meaning in life. The relation between mature happiness and eudaimonia was additionally evidenced by the moderate correlations observed between the MHS-R and PERMA subscale of meaning in life. This also corroborates the ancient but prevailing stoic idea that equanimity or freedom from disturbing emotions (in the sense that they do not exert control over the person's decisions) is a main component of an eudaimonic life (Pigliucci, 2020).

The results mentioned above were observed after controlling for demographic factors and country-level variables, which manifests certain universality of our findings. Our results are in line with previous studies showing that females, younger people, those with a pre-existing mental disorder and lower economic status are the most psychologically affected groups in this pandemic (e.g., Fitzpatrick et al., 2020; Xiong et al., 2020). However, the current study shows that mature happiness (and PERMA) can be an important component of resilience regardless of gender, age, economic status, a previous mental disorder, personal affection by the pandemic, and cultural background. These findings support a global understanding of happiness based on inner harmony that serves as a more adaptive tool during this unprecedented pandemic. As demographic characteristics and the sampling method were not identical across countries, scores in well-being and distress cannot be meaningfully compared. Future cross-cultural studies with more similar samples could gain an additional understanding of the incidence of mature happiness and PERMA levels across the global sphere. An important contribution of this study is that it demonstrates the predictive protective role of mature happiness on psychological distress during the COVID-19 pandemic involving 30 countries from all continents. Another strength of the study is that, for the first time, it compared mature happiness with PERMA in stressful situations empirically. Before this study, the higher adequacy of mature happiness for stressful conditions as compared to other models of well-being had been solely hypothesized (Wong and Bowers, 2018; Wong, 2020).

Overall, the findings of this study indicate that mature happiness seems to add information to the prediction of mental health beyond PERMA. This may be explained in part by the intrinsic consideration of negative emotions into the conceptualization of mature happiness. Rooted in existential positive psychology (Wong, 2011), mature happiness is defined as a positive mental state characterized by inner harmony, calmness, acceptance, contentment, and life satisfaction (Wong and Bowers, 2018). This mental state is the result of living in harmony between positive and negative aspects of living (including unpleasant emotions, personal burdens, and weaknesses). As this study reflects, this approach to well-being can be more appropriate in times of adversity than PERMA, which does not explicitly include the management of negative affect. Rather than focusing on individual success, excitement, and the use of external resources (such as personal relationships and material resources), mature happiness is focused on the use of inner resources and personal maturity, which depend to a lower extent on external circumstances. This harmony-based happiness can be achieved by mental toughness, responsibility (toward oneself and others), life appreciation, mindfulness, meaning in life, and the belief in a better future, which can be cultivated regardless of personal circumstances (Wong, 2020). In an ongoing international project, we have observed that such strategies play a central role in effective coping with the COVID-19 pandemic (Eisenbeck et al., 2021). Mature happiness can be considered as the mental state by-product of this dialectic way of coping with life demands.

The results of the present study should not be taken as a disregard of the PERMA model of well-being. On the contrary, we have observed that this model also predicts to great extent distress during the COVID-19 pandemic. Far from excluding each other, mature happiness can be used to complement PERMA in order to gain a greater understanding of well-being. Mature happiness, as measured by the MHS-R, seems to add unique features of well-being that are not reflected in PERMA. Although the model of PERMA does not explicitly include the relationship with negative emotions and suffering *in* its conceptualization of happiness, in his book “Flourish: A visionary new understanding of happiness and well-being” (Seligman, 2011), Seligman dedicates several sections to the topic of resilience and the treatment of negative emotions. Based on our results, we believe that these elements together with inner harmony should be integrated into the concept of happiness itself. As Seligman (2018) suggests, the PERMA model is open to the incorporation of new elements. Our study highlights that inner harmony may be an essential facet of well-being to be taken into consideration. As times have drastically changed, so our approaches to well-being can be adapted.

Among the limitations of this study is its cross-sectional design with a convenience sample and the overrepresentation of females and students, which limit the generalization of our results and the assumption of causal relationships among variables. The variable “age” was missing in a portion of the US sample. However, this rarely could have had an impact in the global analyses given the big size of the total sample. We also used self-reports online which can be subject to social desirability. Although the general conclusions of this study about the relationship between mature happiness and PERMA seem to be clear, results of invariance testing showed that there might be some cultural differences that can come from many sources (such as construct, method or item differences). As it has been previously discussed, general CFA invariance testing methods have overly restrictive assumptions (e.g., Brown, 2015), especially for cross-cultural data. Lack of invariance is quite expected in cross-cultural studies with such a large number of groups: data show that these types of studies tend to show low invariance levels (e.g., Dong and Dumas, 2020). As the aim of the present paper was to only depict general trends, future studies should evaluate possible differences between different countries and cultures on well-being measured by these two questionnaires. Furthermore, the advantages of mature happiness in the prediction of distress found in this study are limited to the comparison with PERMA. In this sense, it would be of interest to compare mature happiness with other traditional measures of well-being (e.g., Bradburn, 1969; Diener et al., 1985; Watson et al., 1988; Ryff, 1989). Future studies using other methods of evaluation [for alternative candidates see Diener (1994)] could provide additional evidence on the role of inner harmony in well-being and distress. In spite of this, the cross-cultural large sample used for this study and the systematic results found are solid enough to draw the afore-mentioned conclusions.

In summary, our findings highlight the importance of treating mature happiness in policies and interventions aimed to alleviate the negative effects of the current pandemic in people's mental health. Encouraging mature happiness can help people to deepen in their inside, rather than focusing on the outside, and to learn how to live in balance with all aspects of themselves and their circumstances. The paper provides evidence of inner harmony as an essential facet of well-being, particularly in adverse situations such as the global coronavirus pandemic. This way, our study expands the mainstream conceptualization of well-being and happiness by including a holistic harmonic view of well-being that integrates positive and negative aspects of one's life. The results here described call for the integration of mature happiness when evaluating or promoting well-being in future studies and interventions. According to our findings, interventions targeted to promote mature happiness, in comparison with other well-known approaches to well-being such as PERMA, may have a higher protective impact on psychopathological symptoms such as stress and anxiety.

## Data Availability Statement

The raw data supporting the conclusions of this article will be made available by the authors, without undue reservation.

## Ethics Statement

The studies involving human participants were reviewed and approved by Comisión de bioética, Universidad de Almería. Ethics Commission for Research within the Faculty of Psychology and Educational Sciences, Universitatea “Alexandru Ioan Cuza” Din Iasi. Comité de Ética en Investigación, Universidad de Monterrey. Batna 1 University Rectorat. Department of Applied Psychology, Guru Jambheshwar University of Science and Technology. Comissão Nacional De Ética Em Pesquisa. Medizinische Fakultät Ethikkommission, Heinrich-Heine-Universität Düsseldorf. Kutatásetikai Bizottság, Eötvös Loránd Tudományegyetem, Pedagógiai és Pszichológiai Kar. International University of Business Agriculture and Technology (IUBAT). Comitato Etico Della Ricerca Psicologica (AREA 17) Dipartimenti/Sezione di Psicologia - Università di Padova. Institutional Review Board, Lebanese American University. Ethical Review Board COMSATS University Islamabad, Lahore Campus. Komisija za etiko Filozofske fakultete, Filozofska fakulteta, Univerze v Ljubljani. Pathumwan Institute of Technology. Örebro universitet. Rutgers eIRB. Faculty Ethics Committee, University of South Wales. Agri Ibrahim Çeçen Üniversitesi Rektörlügü. The patients/participants provided their written informed consent to participate in this study.

## Informed Consent

Informed consent was obtained from all individual participants included in the study.

## Author Contributions

DC has contributed to this article by coordinating data collection, elaborating the theoretical framework, and writing the manuscript. NE has contributed to this article through the coordination of data collection, data analysis, and writing the manuscript. JAP-E has contributed to this article through a critical revision of the manuscript and the elaboration of the theoretical framework. JG-M has contributed to this article through data collection and the revision of the manuscript. All authors contributed to the article and approved the submitted version.

## Conflict of Interest

The authors declare that the research was conducted in the absence of any commercial or financial relationships that could be construed as a potential conflict of interest.
